# Histone deacetylase inhibitory and cytotoxic activities of the constituents from the roots of three species of *Ferula*

**DOI:** 10.22038/IJBMS.2018.34338.8151

**Published:** 2019-01

**Authors:** Saba Soltani, Gholamreza Amin, Mohammad Hossein Salehi-Sourmaghi, Mehrdad Iranshahi

**Affiliations:** 1 Department of Pharmacognosy, Faculty of Pharmacy, Tehran University of Medical Sciences, Tehran, Iran; 2 Biotechnology Research Center, Pharmaceutical Technology Institute, Mashhad University of Medical Sciences, Mashhad, Iran

**Keywords:** Apiaceae, *Ferula latisecta*, *Ferula ovina*, *Ferula flabelliloba*, Histone deacetylase – inhibitors, Cytotoxic activities

## Abstract

**Objective(s):**

Histone deacetylase inhibitory and cytotoxic activities of 18 naturally occuring terpenoids (ferutinin, stylosin, tschimgine and guaiol), coumarins (umbelliprenin, farnesiferone B, conferone, feselol, ligupersin A, conferdione, conferoside) and sulfur-containing derivatives (latisulfies A-E, persicasulphides A and C) from the roots of three species of *Ferula *(*Ferula latisecta, Ferula ovina* and *Ferula flabelliloba*) were evaluated.

**Materials and Methods:**

The cytotoxic activity of compounds was evaluated against human cancer cell lines (HeLa, HCT116, A2780 and A549) by AlamarBlue® assay using vorinostat as the positive control. On the other hand, we aimed to evaluate their inhibitory activities against pan-HDAC.

**Results:**

The methanolic extract of the roots of *F. flabelliloba* was subjected to silica gel column chromatography. Further purification by preparative thin-layer chromatography (PTLC) and semipreparative RP-HPLC yielded twelve known compounds (1-12). This is the first report on the isolation of guaiol (1), persicasulphide C (3) and conferoside (10) from the roots of *F. flabelliloba*. Six compounds including persicasulfide A, conferone, feselol, latisufide C, conferoside and ferutinin showed cytotoxic activity with IC_50 _values in the range of 11.61-49.40 μM against cancer cells and pan-HDAC inhibitory activity with IC_50_ values in the range of 1.06-35.27 μM.

**Conclusion:**

Results indicated that persicasulfide A (2), conferone (6) and feselol (7) showed moderate cytotoxicity with IC50 values in the range of 11.76-39.24 μM against cancer cells and potent pan-HDAC inhibitory activity with IC_50_ values in the range of 1.06-10.73 μM. Conferone was more active than others with a higher potency for HDAC inhibition (1.06- 1.17 μM).

## Introduction

 Acetylation process is an important post-translational modification that regulated by the activity of two families of enzymes with antagonistic functions: histone acetyl transferases (HATs) and histone deacetylases (HDACs) (1). Recent evidence shows that epigenetics play an important role in the origin, development, and metastasis of cancer, thus providing a novel therapeutic strategy (2). Naturally occurring inhibitors of histone deacetylases are currently finding applications as anticancer agents (3). At present, there are continuing research to find new compounds that inhibit HDAC isoforms and that may be used to treat other types of diseases.

**Figure 1:**
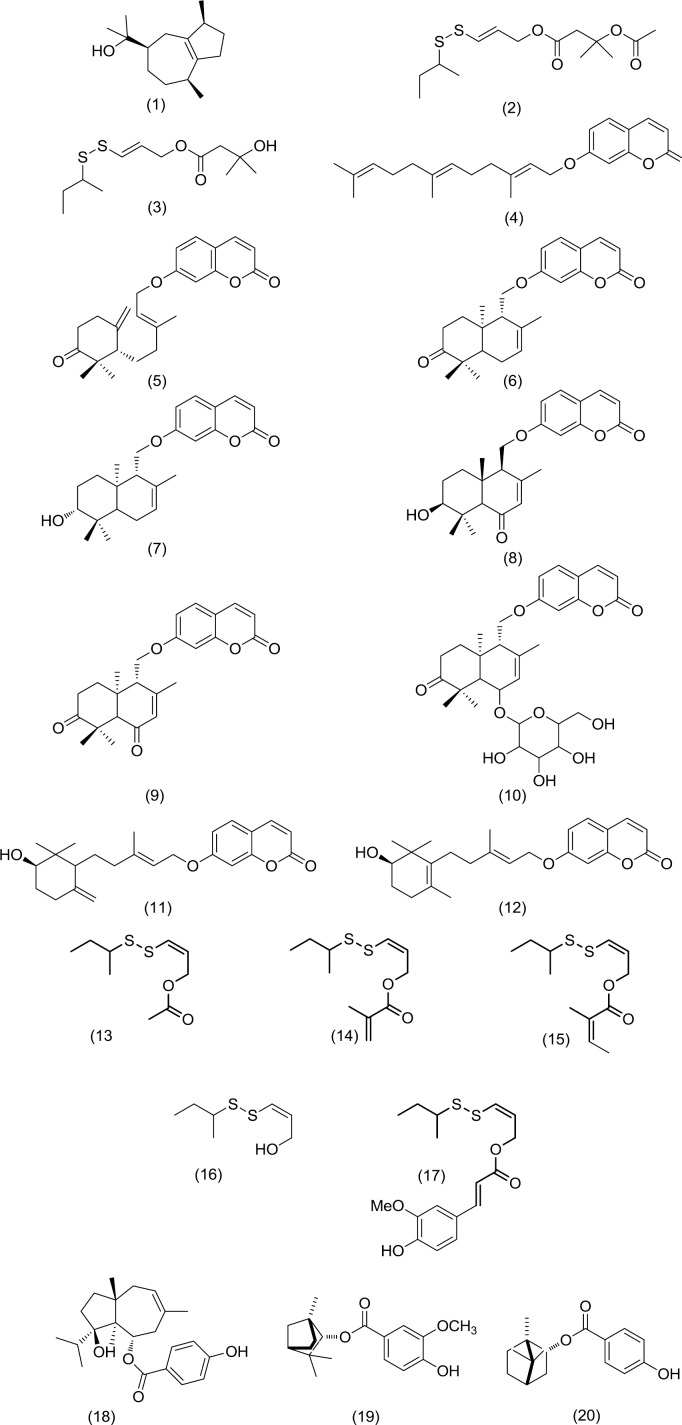
Structures of the isolated compounds from Ferula species

*Ferula* is a large genus of perennial herbs belongs to the Apiaceae family which includes some 180 species (4). The phytochemical analysis of more than seventy *Ferula* species revealed that this genus contains biologically active compounds such as sesquiterpene derivatives and sulfur containing compounds (5-8). Since the last decade, natural products from the genus* Ferula* has been received considerable attention towards the potential biological activities (9, 10). In the present study, five sulfur-containing compounds from our previous study (11), ten isolated compounds from the roots of *Ferula flabelliloba *and three constituents from the roots of *Ferula ovina* (Figure 1), were screened for their cytotoxic activity against four different cancer cell lines that typically overexpress HDAC enzymes, including colon (HCT116), human cervical (HeLa), lung (A549) and ovarian (A2780) cancer cells (12-14). In addition, we evaluated the inhibitory activities of the mentioned natural compounds against pan-HDAC.

## Materials and Methods

***General experimental procedures***

Nuclear magnetic resonance (NMR) spectra were obtained using Bruker AVANCE-300 spectrometer (Bruker, Germany). Chemical shifts are given as δ (ppm) using TMS as an internal standard. Analytical TLC was performed on silica gel 60 F254 (Merck, Germany). Preparative TLC was performed on silica gel 60 GF254 (Merck, Germany). Column chromatography (CC) was conducted with Si gel 230–400 mesh (Merck, Germany). Observation of plates was carried out under UV CAMAG spectrometer (Berlin, Germany) at 254 nm. Semipreparative HPLC was performed on a KNAUER liquid chromatograph system with a quaternary pump (Smartline Pump 1000) and semi-prep c18 column (onyx monolithic; 100×10 mm). Diode array detector (Smartline DAD 2800) and EZ Chrom Elite software was used for detection and processing of data, respectively.

*Plant material*

The roots of *F. flabelliloba* were collected and identified by Dr M Iranshahi, in May 2014, from Khorasan-Razavi province, Golmakan. A voucher specimen (No. 13064) was deposited in the Herbarium of School of Pharmacy, Mashhad University of Medical Sciences.

*Extraction and isolation*

The dried roots of *F. flabelliloba* (250 g) were powdered and extracted three times with MeOH (3×1000 ml, 24 hr each) using maceration method at ambient temperature. TheMeOH extracts were combined and the solvents evaporated under vacuum pressure leaving 50 g of a brown residue. Part of the extract (35 g) was subjected to silica gel chromatography (55×5 cm) using PET: EtOAc and EtOAc- MeOH [(1:0 to 0:1 and 1:1, 0:1, v/v) × 2 L] as a gradient solvent system. Thirty eight fractions were collected and combined into eight major fractions on the basis of their TLC profiles: A [1 – 8; PET: EtOAc (9 : 1); 4.5 g; TLC (Hex: EtOAc 9 : 1.5)], B [9 – 14; PET: EtOAc (9:1 and 8 : 1); 10.4 g; TLC (Hex: EtOAc 9 : 2)], C [15 – 16; PET: EtOAc (8 : 1 and 7:1); 2.43 g; TLC (Hex: EtOAc 9 : 2)], D [17; PET: EtOAc (7 : 1); 2.3 g; TLC (Hex: EtOAc 9 : 2.5)], E [18-21; PET: EtOAc (6:1); 2.0 g; TLC (Hex: EtOAc 9 : 2.5)], F [22 – 24; PET: EtOAc (6:1 and 5:1); 2.2 g; TLC (Hex: EtOAc 9 : 2.5)], G [24 – 30; PET: EtOAc (4:1 and 3:1); 3.2 g; TLC (Hex: EtOAc 9 : 4)], and H [31 – 38; PET: EtOAc (2:1, 1:1 and 0:1), EtOAc: MeOH (1:1, 0:1); 7.2 g; TLC (EtOAc: MeOH 9 : 1)]. Fractions A–D were further purified using PTLC. Finally, compound **1** (10 mg, Rf 0.55) and** 2** (90 mg, Rf 0.44) was obtained from fraction A using DCM system. Compound **3** (12 mg, Rf 0.6) was obtained from fraction C using PET: EtOAc (4:1.5) system. Fraction B afforded off-white crystals of compound **4** (130 mg). Compound **5** (51 mg, Rf 0.37) was obtained from fraction D using DCM: MeOH (10:1) system. Fraction E afforded white crystals of compound **6** (148 mg). Fraction F afforded white crystals as an inseparable mixture of Compounds **11** and **12** (77 mg). Fraction G was separated and further purified by Semipreparative HPLC with a gradient of MeOH–H_2_O on a semi-prep C18 (onyx monolithic; 100×10 mm) column to yield compounds **7** (25 mg, t_R_ 5.48 min), **8 **(15 mg, t_R_ 6.15 min) and **9** (36 mg, t_R_ 6.57 min). Fraction H was subjected to silica gel CC (5 × 80 cm) by gradient polarity of  DCM: MeOH (6 : 1, 3 : 1, 1 : 1) to afford compound **10** [42 mg; TLC (DCM: MeOH 3 : 1, Rf 0.43)]. The structures of the compounds were assigned via comparison of ^1^H- and ^13^C- NMR spectra with those of previous reports (Figures S1-16 and Table S1). Latisufides (A-E) (**13-17**) were isolated and described in our previous report (11). Tschimgine, ferutinin and stylosin (**18-20**) were isolated from the roots of *F. ovina* as previously described (15).

**Table 1:** Cytotoxic activity and HDAC inhibition [IC_50_ (μM)] of compounds 1-20 and vorinostat against human colon cancer (HCT116), human cervical (HeLa), lung (A549) and ovarian cancer (A2780) cell lines

Compound	Cytotoxicity IC_50_				HDAC inhibition	
	HeLa	HCT116	A2780	A549	HeLa	HCT116
Persicasulfide A (2)	11.76 ± 11.58	11.61 ± 10.48	19.38 ± 7.29	15.35 ± 6.77	5.47 ± 0.89	4.41 ± 0.24
Persicasulfide C (3)	64.84 ± 1.01	>100	>100	>100	>70	>70
Umbelliprenin (4)	>100	>100	>100	>100	>70	>70
Farnesiferone B (5)	71.64 ± 6.4	50.1 ± 6.29	78.91 ± 6.74	72.50 ± 4.83	>70	40.35 ± 1.09
Conferone (6)	38.21 ± 7.8	31.77 ± 8.33	32.35 ± 9.83	39.24 ± 5.23	1.17 ± 0.06	1.06 ± 0.15
Feselol (7)	34.76 ± 7.94	28.38 ± 8.1	20.16 ± 9.68	25.52 ± 5.86	10.73 ± 1.31	5.29 ± 0.22
Ligupersin A (8)	22.95 ± 6.82	68.50 ± 7.41	39.50 ± 10.78	33.51 ± 9.58	2.86 ± 1.40	26.44 ± 0.03
Conferdione (9)	41.74 ± 11.07	50.88 ± 5.19	38.83 ± 9.88	32.69 ± 7.96	17.37 ± 0.50	>70
Conferoside (10)	39.95 ± 7.99	35.28 ± 6.98	36.38 ± 12.32	45.77 ± 6.39	35.27 ± 0.16	9.94 ± 1.89
Latisulfide A (13)	77.63 ± 3.43	>100	>100	>100	>70	>70
Latisulfide B (14)	>100	>100	>100	>100	>70	>70
Latisulfide C (15)	18.69 ± 8.5	47.17± 8.18	49.40 ± 6.2	47.93 ± 4.73	10.59 ± 0.77	25.91 ± 0.14
Latisulfide D (16)	>100	>100	>100	>100	>70	>70
Latisulfide E (17)	30.64 ± 14.91	57.08 ± 5.21	71.29 ± 6.52	69.66 ± 7.71	5.02 ± 0.29	19.92 ± 0.69
Ferutinin (18)	35.31 ± 8.40	27.03 ± 9.01	21.27 ± 7.18	25.03 ± 13.95	33.39 ± 0.18	1.45 ± 0.10
Stylosin (19)	54.78 ± 7.53	42.08 ± 6.1	25.52 ± 5.96	29.69 ± 10..29	>70	14.93 ± 0.08
Tschimgine (20)	54.80 ± 7.38	34.89 ± 4.71	45.44 ± 9.88	56.33 ± 11.58	>70	0.56 ± 0.38
Vorinostat	2.43 ± 5.02	2.98 ± 1.02	8.02 ± 9.8	6.83 ± 6.6	0.06 ± 0.10	0.05 ± 0.13

*Cell culture*

Cervical (HeLa), ovarian (A2780), and lung (A549) cancer cells, obtained from Biotechnology Laboratory stocks, Biotechnology Research Center (Mashhad, Iran) and colon (HCT116) obtained from National Cell Bank of Iran (NCBI), Pasteur Institute of Iran, Tehran (Iran). All cells were grown in Roswell Park Memorial Institute (RPMI)-1640 supplemented with 10% fetal bovine serum (Paisely, UK), penicillin (100 units/ml) and streptomycin (100 mg/ml) in an atmosphere of 95% O_2_ and 5% CO_2_ at 37 ^°^C.

*Cytotoxicity assay*

*In vitro* cytotoxicity tests were performed using a nonfluorescent substrate, AlamarBlue® (Paisely, UK) cell viability assay as described with some modifications (16). Four cell lines (HCT116, HeLa, A549 and A2780) were grown in RPMI-1640 medium at 37 ^°^C in a 5% CO_2_ incubator. After counting, the cells were seeded in 96-well microplates (1 × 10^4^ cells/100 µl in each well) and cultivated for 24 hr before addition of test compounds. Triplicate plates for three wells of each concentration were seeded. The test compounds, were dissolved in dimethyl sulfoxide (final concentrations of DMSO per well was 0.1% V/V). Then, the cells were treated with the different concetrations of the test compounds (100, 50, 25, 12.5, 6.25 μM), positive control, negative control and cell culture medium as blank for 48 hr in a 5% CO_2_ incubator. Thereafter, 10 μl of AlamarBlue® reagent was added and incubated for another 4 hr. Then, absorbance was measured at 600 nm using an ELISA microplate reader (Epoch; USA). IC_50_ values were calculated by nonlinear regression analysis using GraphPad prism (Version 6.0) software. Positive control was vorinostat (purity ≥98%; Sigma-Aldrich, USA).

*Whole-cell HDAC inhibition assay*

The HDAC assay was based on an assay by Marek *et al.* (17) with minor modifications. Briefly, HeLa and HCT116 cells, were seeded in 96-well microplates (1.5 × 10^4^ cells/90 µl culture medium per well). Three wells defined as 100% initial activity and three wells defined as background and three wells for each concentration were seeded. After 24 hr, the cells were further incubated for 18 hr with increasing concentrations of test compounds, positive control vorinostat and cell culture medium alone as negative control at 37 °C with 5% CO_2_. The test compounds, were dissolved in dimethyl sulfoxide (final concentrations of DMSO per well was 0.1% V/V) and their concentrations adjusted to 70, 40, 20, 5, 1 μM through dilution with the growth medium. The first step of reaction was started by adding 10 µl of 3 mM Boc-Lys (ε-Ac)-AMC (Bachem, Switzerland) to each well to reach a final concentration of 0.3 mM. Then, cells were incubated with the Boc-Lys (ε-Ac)-AMC for 3 hr under cell culture conditions. After this, 100 µl/well of stop solution (25 mM Tris-HCl (pH 8), 137 mM NaCl, 2.7 mM KCl, 1 mM MgCl_2_, 1% NP40, 2.0 mg/ml trypsin, 10 µM vorinostat) was added and the mixture was developed for another 3 hr under cell culture conditions. The final volume of the solution (210 µl per well) were transferred to the black 96-well microplate with flat clear bottom. We calculated fluorescence intensity at an excitation of 360 nm and emission of 470 nm in a NOVO star microplate reader (BMGLabTech, Germany).

*Trypsin inhibitory assay*

Trypsin inhibition assay was carried out based on the method of Zwick *et al.* (18) with some modifications. Reactions were set up in 96-well microplates in triplicate. 2.0 mg/ml of trypsin and vorinostat (10 μM) were dissolved in assay buffer (25 mM Tris at pH 8.0 adjusted with HCl, 137 mM NaCl, 2.7 mM KCl, 1 mM MgCl_2_) and added (except for blanks) to each microplate well. Test compounds were dissolved in DMSO and added at a final concentration of 70 μM (1% DMSO) in each well. The reaction initiated when BOC-Lys-AMC substrate (Bachem, Switzerland) was added. Then, plate was incubated for 45 min. The amount of trypsin inhibition was calculated by comparing the amount of deacetylated substrate between control and test compounds. We calculated the relative amounts of deacetylated substrate by fluorescence reading with excitation at 360 nm and emission at 470 nm. (Caffeic acid was as positive control).

## Results

The MeOH extract from the roots of *F. flabelliloba *were subjected to silica gel column chromatography. Further purification for collected fractions developed by the preparative thin-layer chromatography (PTLC) and semipreparative RP-HPLC yielded twelve known compounds (**1-12**). A sesquiterpenoid alcohol, guaiol (**1**), two sulfur-containing compounds, persicasulphide A (**2**) and C (**3**), the prenylated coumarin umbelliprenin (**4**), five sesquiterpene coumarins, farnesiferone B (**5**), conferone (**6**), feselol (**7**), ligupersin A (**8**) and conferdione (**9**) and inseparable mixture of farnesiferol B and lehmferin (11,12) together with a sesquiterpene coumarin glycoside, conferoside (**10**).

The structures of the mentioned known compounds **1-12** (Figure 1) were in agreement with the literature (6, 19, 20) and our in-house NMR bank. This is the first report of compounds 1, 3 and 10 from the roots of *F. flabelliloba*.

As shown in Table 1, the cytotoxicity activity of 18 natural compounds was evaluated against HeLa, HCT116, A2780 and A549 cells by AlamarBlue® assay using vorinostat as the positive control. The IC_50_ values were in the range of 11.61-78.91 μM against all cell lines. *In vitro* pan-HDAC inhibitory activity was evaluated on HeLa and HCT116 cell lines. (Vorinostat as the reference drug, Table 1).

Our findings revealed that six tested compounds persicasulfide A (**2**), conferone (**6**), feselol (**7**), latisulfide C (**15**), conferoside (**10**) and ferutinin (**18**) possessed moderate cytotoxicity against the cancer cell lines with IC_50_ values in the range of 11.61-49.40 μM. These compounds showed pan-HDAC inhibitory activity with IC_50_ values in the range of 1.06-35.27 μM. The most potent cytotoxic test compound was persicasulphide A (**2**) with IC_50_ values of 11.61, 11.76, 15.35 and 19.38 μM against HCT116, HeLa, A549 and A2780 respectively. The test compounds were subjected to an* in vitro *trypsin inhibition assay to test for interference with the assay by direct trypsin inhibition. According to this assay, 13 compounds were evaluated at a maximum concentration of 70 μM and did not display any inhibition toward trypsin (Figure S18). These results suggested that the HDAC inhibition measured was not due to the protease inhibition and the fluorescence-based assay clearly reflect the real HDAC activity.

## Discussion

The “alkylthio” moiety represented by S-alkyl-S-alkenyl disulfides in persicasulphide A (**2**), latisulfide C (**15**) and latisulfide E (**17**) probably responsible for their biological activities via disulfide exchange mechanism. Among sulfur-containing derivatives, persicasulphide A from *F. flabelliloba* showed the most HDAC inhibition and cytotoxic activities. Disulfides as thiol oxidizing agents, able to  oxidize thiolate cysteine residues on proteins to form mixed disulfides, thereby affecting protein or enzyme function and leading to cell death (21). Shokoohinia *et al.* (22) found that, thiol exchange would lead to the thioacrolein in the case of asadisulfide-type esters. This activity was previously observed in similar sulfur compounds including isothiocyanates from mustard oil and sulfonates from onion and garlic (23). Metabolic conversion of organosulfur compounds of garlic and other *Allium* vegetables to HDAC inhibitors *in situ* (allyl mercaptan, allyl methyl sulfide, allyl methyl sulfoxide, allyl methyl sulfone, S-allylmercaptocysteine) may contribute to the cancer chemoprotective properties (24). Nian *et al* . (25) screened garlic organosulfurs and identified allyl mercaptan as the most potent HDAC inhibitor in assays with HeLa nuclear extracts and purified human HDAC8. 

Among coumarins tested, conferone (**6**) (Figure 1) was the most potent HDAC inhibitor with IC_50_ values of 1.06 and 1.17 μM, in HCT116 and HeLa cell lines, respectively. This drimane-type sesquiterpene coumarin ether compound also showed moderate cytotoxicity against all cells with IC_50_<50 µM. Since the coumarin moiety of sesquiterpene coumarins farnesiferone B (**5**), conferone (**6**), feselol (**7**), ligupersin A (**8**), conferdione (**9**) and conferoside (**10**) were identical; differences in cellular effects were most likely due to the presence of hydrogen bond, arene-arene and hydrophobic interactions that may be specific to active site of the histone deacetylase enzyme. Previous investigations revealed a cytotoxic effect of conferone on human colorectal adenocarcinoma, ovarian carcinoma, lung cancer, melanoma and renal cell lines (26-28). In these studies, conferone alone did not show any considerable cytotoxic activity. Cheraghi *et al.* (28) showed conferone could induce cytotoxicity activity by apoptosis in HT-29 cells. They have also demonstrated that conferone could induce anti-angiogenic properties by inhibiting *in vitro* tubulogenesis and reducing the secretion of pro-angiogenic factors. Their study also showed an inherent potency of conferone to bind P-glycoprotein (Pgp) (28). This affinity is in agreement with the observation that a binding site for sesquiterpenes exists within the transmembrane domain of Pgp. Some experiments showed that conferone enhanced the cytotoxicity activity of vinblastine in MDCK-MDR1 cells and vincristine against 5637 cells, these results can be explained by the fact that conferone can probably block Pgps by attachment to these pumps, which results in accumulation of vincristine and vinblastine inside the cells (26). In addition, previous investigations (29-31) have shown that Pgp contributed to the resistance mechanism against some HDAC inhibitors (FK228 and apicidin) since they were substrates of the efflux pump. Our current study indicating that, despite the significant HDAC inhibition after 18 hr treatment with rather wide coumarins (conferone and feselol) concentration, no apparent cytotoxicity was achieved after 48 hr treatment. A possible explanation would be that they may suffer Pgp efflux and not accumulate in the cell over time. 

Peptidase inhibitory activity of test compounds may be considered as one of the main explanation for the relatively potent HDAC inhibition of compounds in contrast to their less pronounced cytotoxicity. Although, results could be affected by this effect and numerous natural products are already known to inhibit trypsin (32, 33), our results was not due to the protease inhibition and the fluorescence-based assay clearly reflect the real HDAC activity.

## Conclusion

In the present study persicasulfide A (**2**), conferone (**6**) and feselol (**7**) showed moderate cytotoxicity with IC_50_ values in the range of 11.76-39.24 μM against cancer cells and potent pan-HDAC inhibitory activity with IC_50_ values in the range of 1.06-10.73 μM. Conferone was more active than others with a higher potency for HDAC inhibition (1.06- 1.17 μM).

## Associated content

**Supporting Information**

NMR spectra and spectral data for all compounds tested in this study are shown in Figure S1- 16 and Table S1. Column graphs of dose-dependent growth inhibition are shown in Figure S17. Trypsin inhibition of test compounds in the trypsin inhibitory assay are shown in Figure S18.

## Conflicts of Interest

The authors declare that there are no conflicts of interest.
